# Supplementary material for: Ordering, flexibility and frustration in arrays of porphyrin nanorings
Source: Nat Commun. 2019 Jul 3;10:2932. doi: 10.1038/s41467-019-11009-y (PMC6610075; doi:10.1038/s41467-019-11009-y)
Supplement: Supplementary file 1 — Supplementary Information [file 41467_2019_11009_MOESM1_ESM.pdf]

Ordering, Flexibility and Frustration in Arrays of Porphyrin Nanorings

Summerfield et al.

1. Supplementary Figures

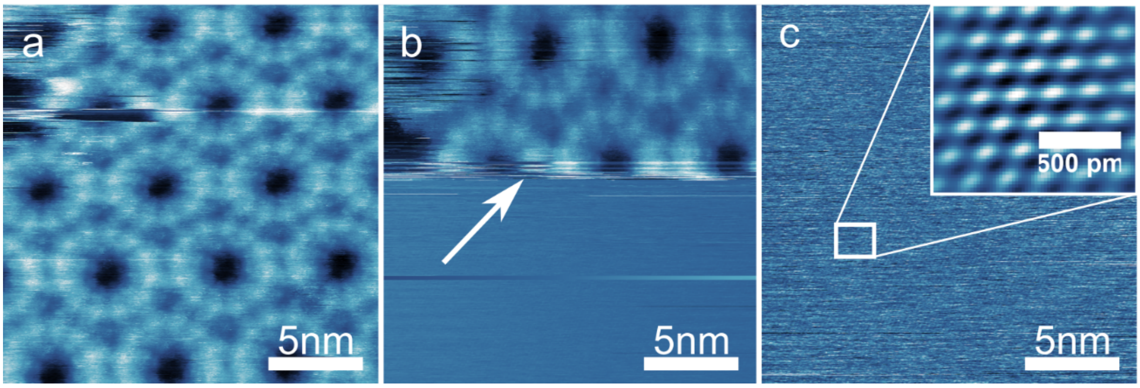

Supplementary Figure 1. Sequential 20 x 20 nm STM images of c-P12 on HOPG showing the conformation of the c-P12 lattice with the graphite substrate by switching imaging modes. a) Up scan of c-P12 domain. b) Down scan of c-P12 lattice and underlying HOPG, the arrow shows the point during the scan when the tip voltage was dropped to 0.1 V and the current set point raised to 1 nA to image the underlying graphite lattice. c) Final up scan of the HOPG lattice with a Fourier filtered section of the region indicated by the white box (inset) showing the hexagonal HOPG lattice.

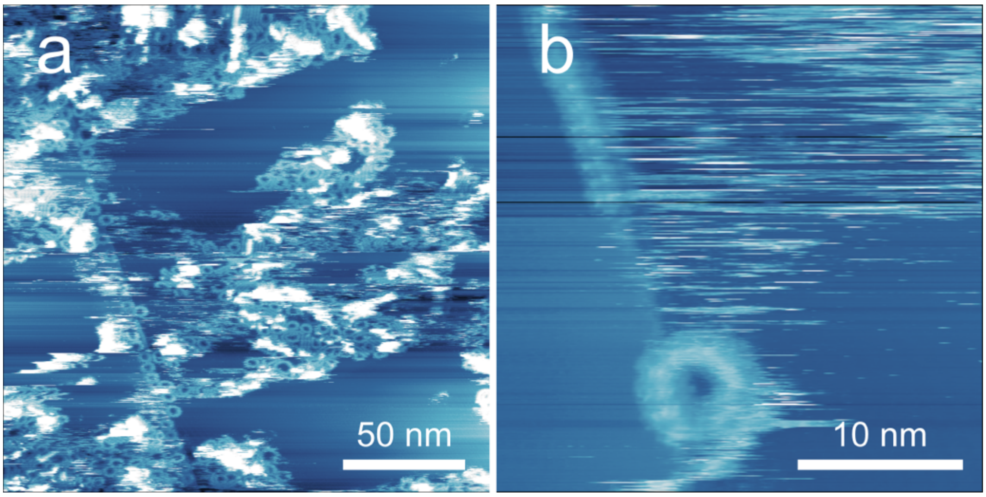

Supplementary Figure 2. STM images of an analogue *c*-P12 adsorbed on HOPG. This analogue of *c*-P12 incorporates teritary butyl solubilising groups[1] (as opposed to alkoxy solubilising groups which were used in the experiments described in the main paper). Under similar experimental conditions only small numbers of nanorings were observed and these were typically adsorbed at step edges on the HOPG substrate. The arrays of this analogue *c*-P12 were highly unstable making extended imaging unfeasible. This figure shows some of the few images of this species in which nanorings could be resolved.

2. Supplementary Methods

To explore the morphology of aggregates of *c*-P10 and *c*-P12 nanorings on graphite, classical molecular dynamics (MD) simulations have been performed using the LAMMPS simulation package[2]. The well-established OPLS[3,4] potential has been employed with additional parameters derived from density functional theory (DFT) calculations. Most of the OPLS parameters for the zinc-porphyrin monomer can be found in the general OPLS database, however for the aromatic core and lateral groups, some parameters are not included in the database. For the aromatic core, the parameters for the Lennard-Jones (LJ) potential and bonding terms (bonds, angles dihedrals and impropers) were inferred from pyrrole. Parameters for the lateral groups are based on anisole and standard alkane chains. To reduce the computational cost of the DFT calculations, the lateral alkane chains were partially removed from the monomer leaving only one CH_2 and CH_3 groups. A monomer, dimer and trimer have been initially optimised using density functional tight-binding (DFTB) formalism[5] as implemented in the DFTBT+ program package[6]. The 3ob-3-1 Slater-Koster parametrisation set[7] for organic and biological systems was employed. The monomer and dimer geometries were subsequently refined using DFT, as implemented in the CP2K program package[8], with tighter convergence criteria (Supplementary Figure 3a).

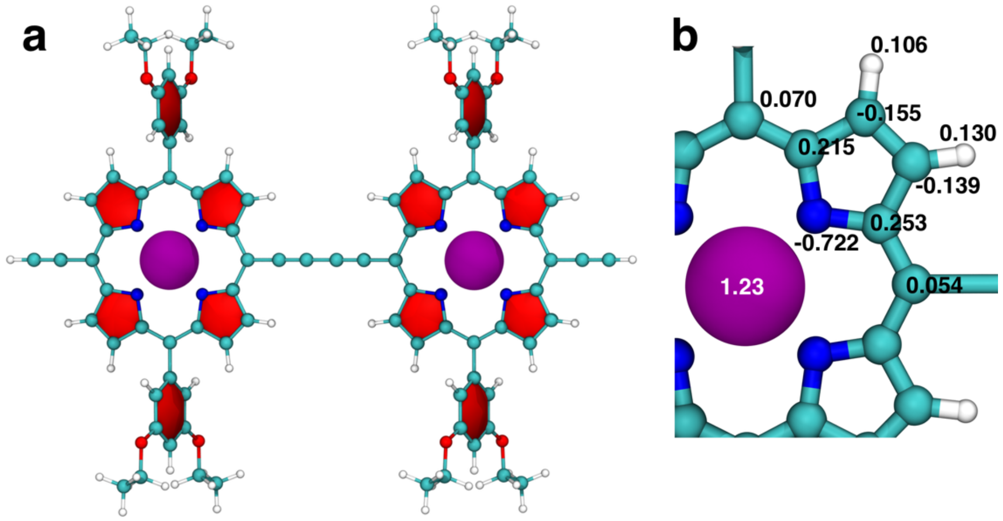

Supplementary Figure 3. a) DFT optimised geometry of a porphyrin core dimer. b) Calculated RESP charges for the porphyrin aromatic core. Zn atoms are highlighted in van der Walls representation.

DFT calculations were performed at the gradient-corrected level by applying the BLYP[9,10] exchange-correlation functional within the GPW approach[11]. Electronic states were expanded by a double-ζ plus polarisation basis set, DZVP,[12] with norm-conserving pseudo-potentials for the description of core levels[13–15] and a plane-wave representation of the electron density in real space with a cut-off of 500 Ry. The atomic charges were calculated on the monomer using the restrained electrostatic potential (RESP) scheme[16] based on DFT. Identical types of atoms were constrained to have the same charge. The charges of atoms outside the aromatic core were restricted to their standard OPLS values. The charge of Zn atom was also constrained to its OPLS value. The Martyna-Tuckerman Poisson solver[17] was used in combination with a relatively large simulation box (up to 3.0 nm in the main dimension) to avoid computational artefacts. The obtained set of charges are shown in Supplementary Figure 3b, which and are in a good agreement with results previously obtained for similar system[18].

Two new OPLS atom types (highlighted by red and yellow circles in Supplementary Figure 4) were introduced to achieve a better match with the DFT geometry. These are based on aromatic carbon type with modified parameters for equilibrium values of bonds and angles shown in Supplementary Figure 4. While the aromatic core of porphyrin monomer is essentially rigid, the polyyne chain linkers are flexible and account for the overall flexibility of the circular polymers. The force constants for the angles involving alkyne

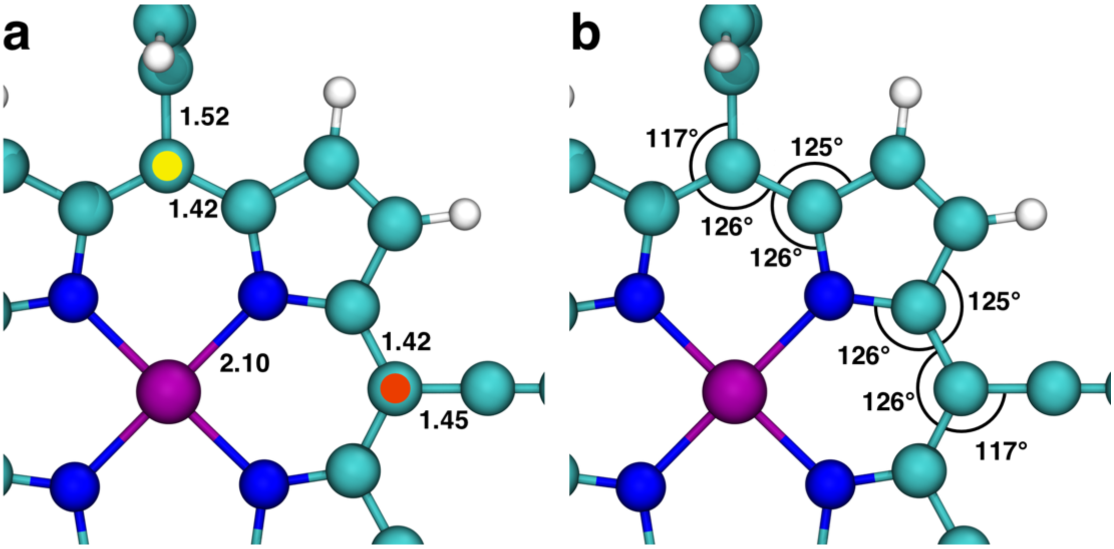

Supplementary Figure 4. The values of equilibrium bond distances (a) and angles (b) involving two new types of OPLS aromatic carbon atoms highlighted by red and yellow circles.

chains were estimated via a series of single point DFT calculations in which the angles highlighted in colours in Supplementary Figure 5a were artificially bent.

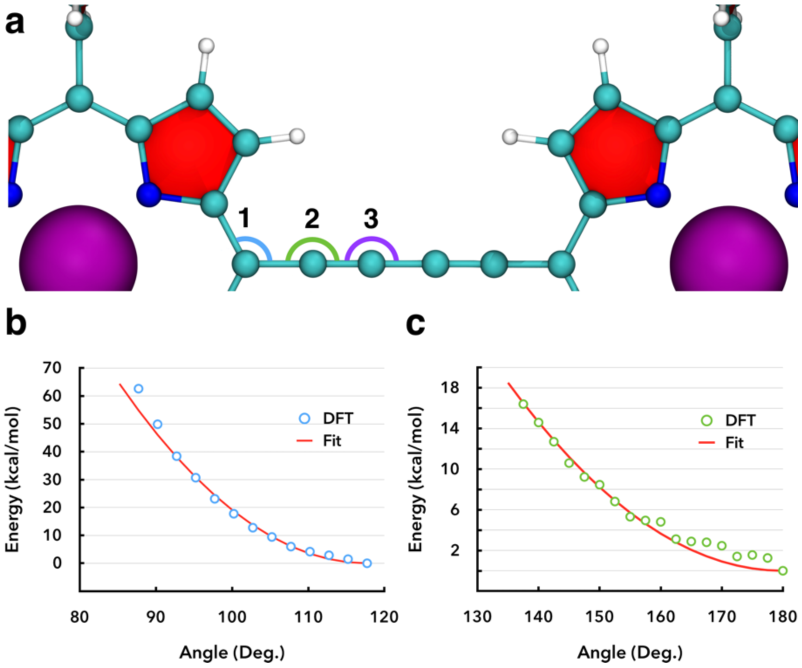

Supplementary Figure 5. a) Angles considered in the fitting procedure. b,c) calculated DFT energy as a function of the depicted angles and the fitting with harmonic angle functions. Angle depicted in purple gives the same results as that shown in c).

The difference in energy compared to the equilibrium geometry was fitted with a harmonic angular function as implemented in LAMMPS (see Supplementary Figure 5b and c). The obtained force constants are 100 kcal/mol/radian^2 for angle 1, and 30 kcal/mol/radian^2 for angles 2 and 3, as shown in Supplementary Figure 5a. Please note that there are two equivalent angles 1 acting at the same time. As previously noted in the literature[19], there is a torsional barrier for the rotation of a porphyrin monomer around the chain. While this could be crucial for the description of the conformations of porphyrin chains in solution, we could avoid introducing additional torsional parameters given the surface confinement of the flat rings. All the obtained parameters were collected to build the models and potential for nanorings of any size (see, for example, c-P10 and c-P12 structures in Supplementary Figure 6 a,b) using VMD[20], TopoTools[21] and Moltemplate[22].

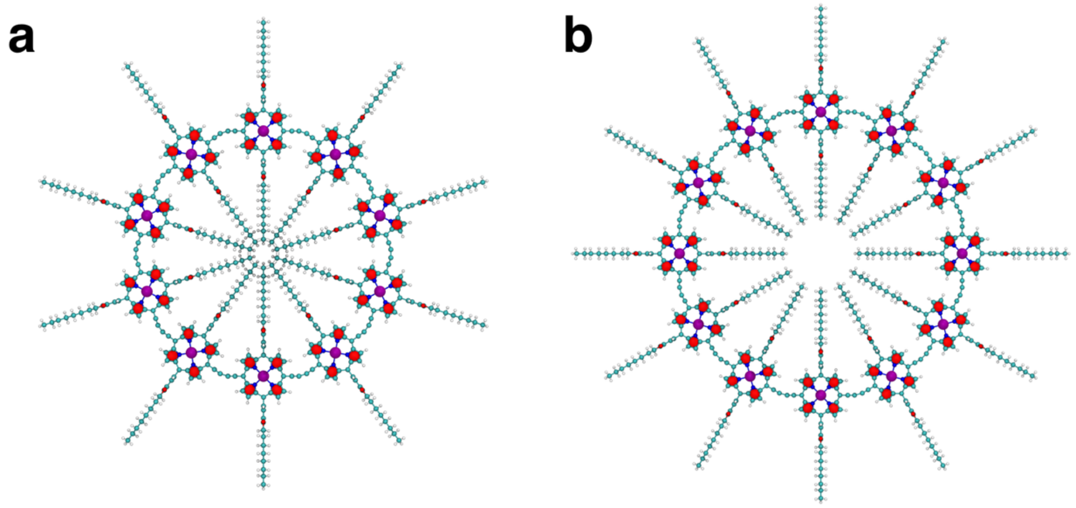

Supplementary Figure 6. Atomistic models of a) c-P10 and b) c-P12 nanorings used in MD simulations.

A series of nanoring models ranging from *c*-P8 to *c*-P30 were built without the lateral alkane chains. The structures were minimised in vacuum with tight convergence criteria and the total energy along with its partial contributions was evaluated. Collected data are shown in Supplementary Figure 7. Both the total potential energy and the mechanical energy are linearly proportional to $1/n^2$ (where n is the number of monomers in the ring) with a gradient related to the overall bending coefficient of nanorings. These data were used to setup the coarse-grained force field for larger scale simulations.

In order to discriminate between the different aggregates of *c*-P10 and *c*-P12 on graphite, MD simulations have been performed on arrays containing 56 nanorings on a graphitic surface. The graphite surface was represented by a square model of a single layer graphene with dimension of 60 × 60 nm with periodic boundary conditions (PBC). PBC in 3 dimensions were used for the simulation of the 2D systems, by inserting a vacuum region of about 4.0 nm along the *z*-direction. A "frame" of Carbon atoms (8 Å width) at the boundary of the simulation box were kept frozen during the MD runs by excluding them from the integrators. This allows us to avoid

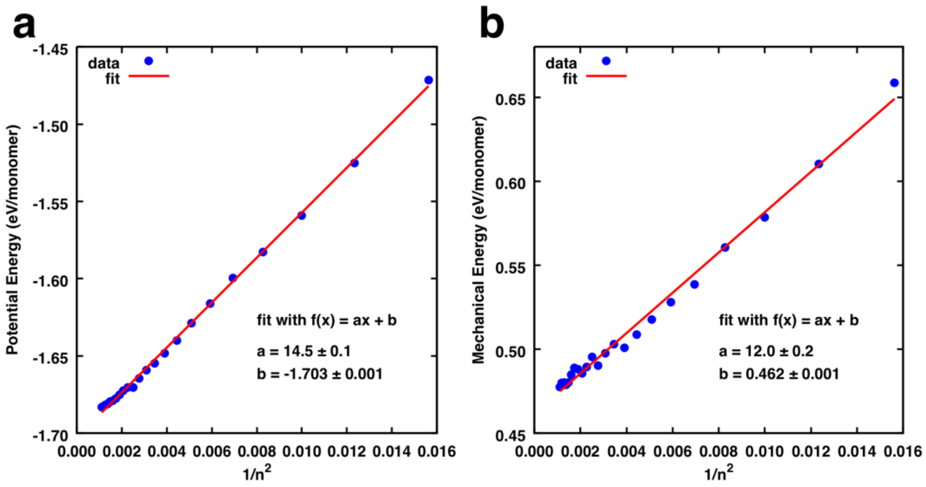

Supplementary Figure 7. a) Total potential energy and b) mechanical energy contribution for nanorings of increasing size.

unphysical oscillation and to resemble closely the behaviour of graphitic surface while keeping the number of C atoms to minimum (140,000). Carbon atoms were parametrised using the OPLS naphthalene fusion carbon. Specific LJ parameters for graphene[23] were tested leading essentially to the same qualitative results. MD simulations have been performed with a time step of 1 fs. The systems have been initially equilibrated using the Nosé–Hoover thermostat[24–26] with a time constant of 0.1 ps for 5 ns and then propagated in the NVE ensemble in excess of 200 ns. While the total energy proved to be very stable, every 40 ns a short (100 ps) NVT run has been performed to compensate for the heat released during nanorings relaxation. Long-range electrostatic interactions

were treated by the particle-particle particle-mesh method with the relative error in electrostatic forces of 10^{-5}. A cut-off distance of 1.2 nm was used for both Coulomb and van der Waals interactions. The starting geometries of the nanorings were obtained by equilibrating a single ring on a small graphene surface for 20 ns at room temperature. The structures have been replicated in hexagonal arrays with large spacing as shown in Supplementary Figure 8. Each model includes 56 nanorings for a total number of atoms up to 250,000.

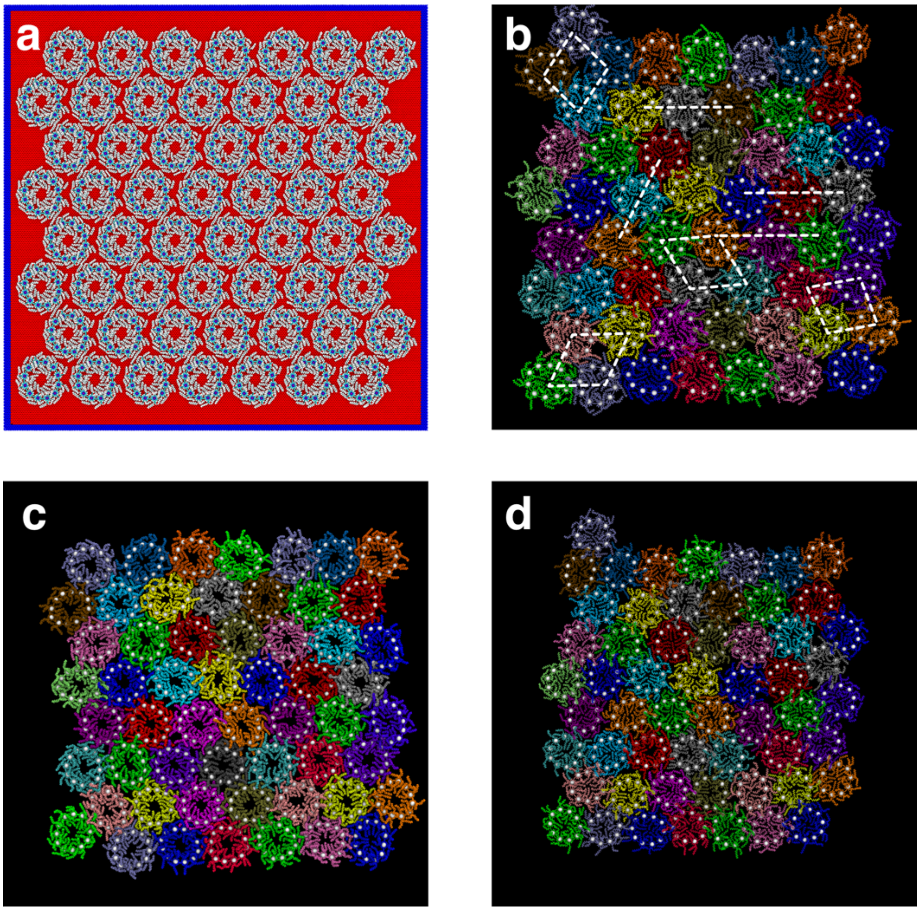

Supplementary Figure 8. a) Example of starting geometry for *c*-P12 aggregate. b) Snapshot from an atomistic MD run of *c*-P10 aggregate equilibrated at 300K; some of the well-formed rhombic and row-like packing features are highlighted with dashed white lines. c-d) Snapshots from atomistic MD runs of c) *c*-P10 and d) *c*-P12 nanorings annealed at 400K.

For quantifying the local ordering of the two aggregates, the 2D radial distribution function of the centre of mass of Zn atoms of each nanoring has been evaluated in both cases. As shown in

Supplementary Figure 9a, *c*-P12 has a very sharp first peak while *c*-P10 exhibits a broader and less structured peak. For *c*-P12 we observe two peaks in the second shell whereas for *c*-P10 they coalesce in a broad shoulder pointing to a less ordered aggregate at longer range. The binding energy between the neighbouring rings in *c*-P12 aggregate has been extracted from classical molecular dynamics simulations performed using the LAMMPS simulation package[2] and averaged over a time period of 5 ns resulting in a distribution with a peak around 1.2 eV (Supplementary Figure 9b). A substantial contribution to the nanoring-nanoring binding energy, up to about 30%, comes from the direct interaction between alkane chains. Supplementary Figure 9c shows that for c-P12 aggregates the interaction energy between alkane chains residing on adjacent nanorings (0.24 eV - 0.48 eV) is comparable to the interaction of an alkane chain with the substrate through van der Waals interactions (0.34 eV per chain). We have tested the stability of the obtained equilibrium configurations by annealing both the *c*-P10 and *c*-P12 structures at 400K for at least 50 ns. Snapshots taken from MD runs show that the *c*-P12 aggregate (Supplementary Figure 8d) remains substantially unchanged whilst the *c*-P10 structure (Supplementary Figure 8c) exhibits a continuous reconstruction, in agreement with observations at room temperature.

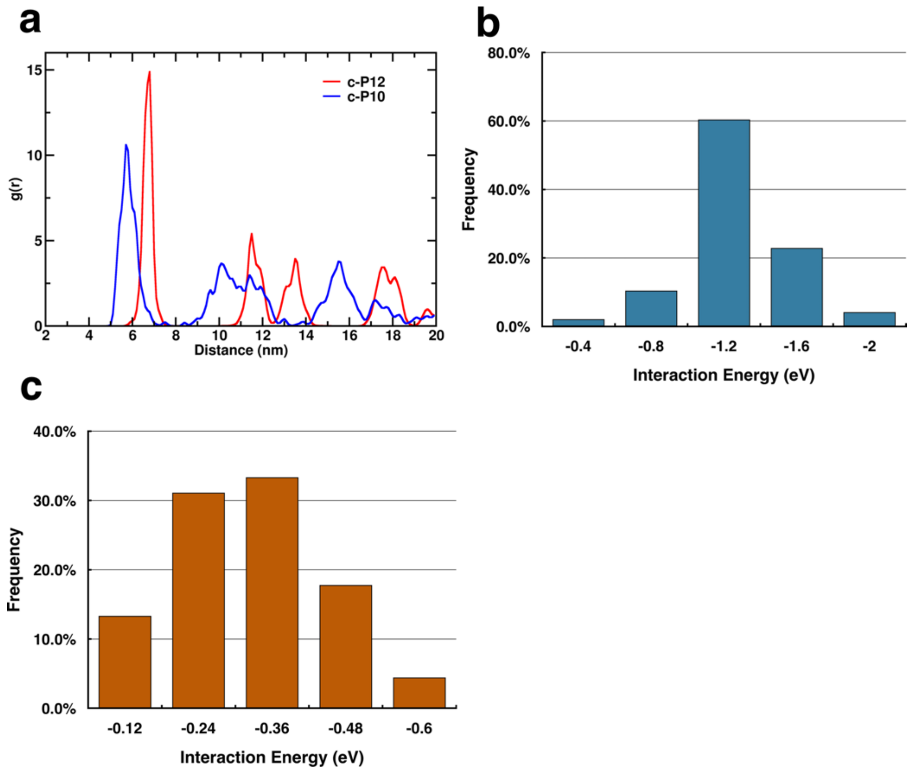

Supplementary Figure 9. a) 2D radial distribution for the centre of mass of zinc atoms in the atomistic MD simulations of *c*-P10 (blue) and *c*-P12 (red) aggregates. b) distribution of the interaction energy between neighbouring nanorings for *c*-P12 aggregate. c) contribution to the total binding energy of *c*-P12 aggregate from the interactions between alkane chains.

The time scale and system size accessible to atomistic simulation impede the observation of larger scale effects. We employ CG simulations to explore the packing of larger aggregates. The well-established MARTINI CG potential has been used[27,28]. MARTINI force-field is a workhorse for CG simulations of biological materials, but it has also been successfully applied to the study of molecular adsorption on graphite[29]. Beads assignment follows Refs. 28-30 as shown in Supplementary Figure 10. The charge of the SQ0 bead, representing

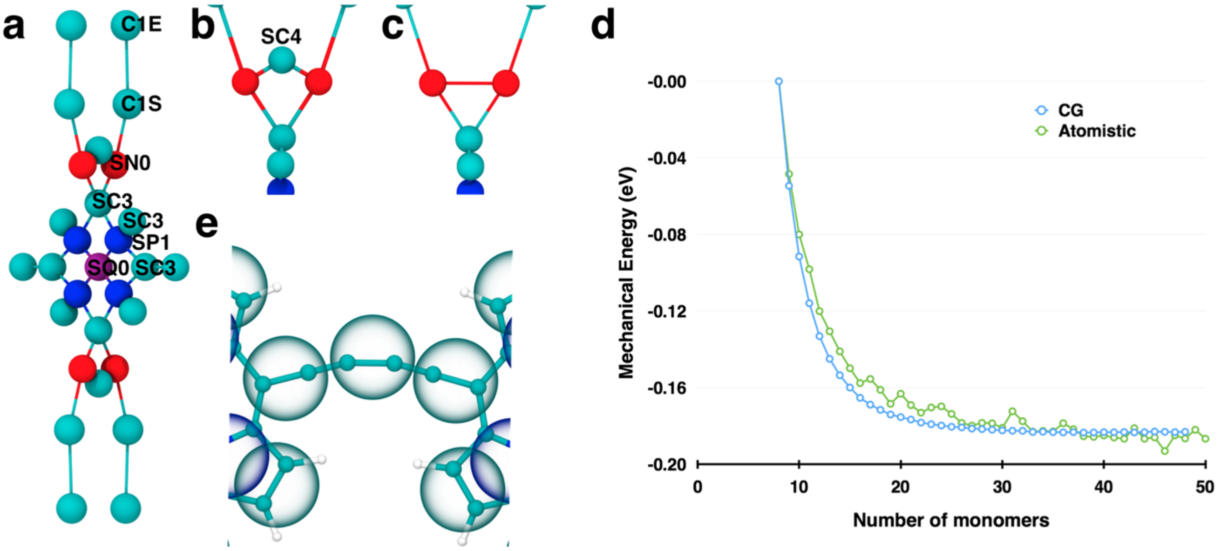

Supplementary Figure 10. a) assignment of MARTINI beads in CG model of porphyrin monomer; b,c) Proposed geometries for the anisole group. d) Comparison between atomistic (red) and CG (blue) mechanical energy contributions for nanorings with an increasing number of monomers. e) Linker beads mapping to atomistic structure between neighbouring nanorings for *c*-P12 aggregate.

the Zn atom, is +0.4 e while that of the SP1 bead representing the azo-group is -0.1.[30] While charges are important for a correct simulation of the rings in solution and in stack arrangement, they don't seem to play a crucial role in our case because of the nature of the surface. As in the case of all-atom models, the porphyrin core is essentially rigid, and all the bonds and angles are stiff. During equilibration this part of the ring have been propagated as independent rigid bodies[31] in order to increase the time-step up to 15 fs while the polyyne linkers and the lateral chains are integrated in a normal NVT ensemble. For the anisole rings two different geometries have been tested as shown in Supplementary Figure 10 b,c. In case (c) the apical CH group has not been mapped, while in (b) it is mapped to a SC4 bead. The latter leads to a much better match in the inter-ring distances, but its size should be decreased to take into account a 1-to-1 mapping. No substantial qualitative difference has been observed between aggregates obtained with the two different mapping schemes. The mapping of the polyyne linker is shown in Supplementary Figure 10 and is similar to a recently

published scheme[32]. As in the case of atomistic simulations, the calibration of bond and angle constants of the polyyne linkers is crucial to reproduce the flexibility of the rings.

A series of CG rings with the increasing number of monomers has been built and optimised, as discussed previously for the atomistic models. The stretching and angular constants between the beads in the monomers and linkers has been tuned in order to match the mechanical energy profile of the atomistic case (see Supplementary Figure 10d). The graphene surface has been modelled using the SG4 special bead introduced in Ref. 29 Not all interactions between the required beads and SG4 were tested in the original paper, but they seem to work correctly, and the thermodynamic data required for the calibration are not easily accessible by experiments for the system of interest. A square model of graphene of 120 x120 nm has been created and up to 224 nanorings positioned on the surface in similar way to that used in atomistic simulations. Models include up to 305,000 beads corresponding to roughly 890,000 atoms. Different starting structures with various initial spacing between the rings have been tested leading to essentially the same qualitative results. The systems have been equilibrated in a mixed rigid and normal integrator, as discusses above, for 200 ns and then fully propagated in an NVT ensemble. Total simulation time exceeds 400 ns. It is important to point out that, because of the smoother nature of the underlying energy landscape, interpretation of time is not straightforward in the CG simulations and the dynamics can be from 2- to 10-fold faster[27].

As in the case of the atomistic model, the radial distribution function on Zn atoms centre of mass has been also evaluated for both nanorings in the CG simulations (see Supplementary Figure 11). In both cases the first peaks perfectly match the experimental values. C-P12 show very sharp peaks almost resembling a crystalline structure whereas *c*-P10 is consistent with various domains of different morphology.

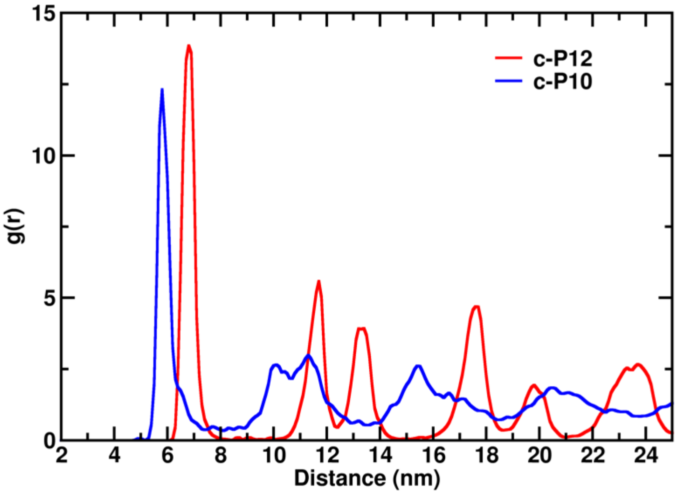

Supplementary Figure 11. Radial pair distribution function of the centre of mass of Zn atoms for *c*-P10 (blue) and *c*-P12 (red) aggregates during GC simulations.

References

1. O'Sullivan, M. C. *et al.* Vernier templating and synthesis of a 12-porphyrin nano-ring. *Nature* **469,** 72–5 (2011).

2. Plimpton, S. Fast parallel algorithms for short-range molecular dynamics. *J. Comput. Phys.* **117,** 1–19 (1995).

3. Jorgensen, W. L. & Tirado-Rives, J. The OPLS [optimized potentials for liquid simulations] potential functions for proteins, energy minimizations for crystals of cyclic peptides and crambin. *J. Am. Chem. Soc.* **110,** 1657–1666 (1988).

4. Jorgensen, W. L., Maxwell, D. S. & Tirado-Rives, J. Development and Testing of the OLPS All-Atom Force Field on Conformational Energetics and Properties of Organic Liquids. *J. Am. Chem. Soc.* **118,** 11225–11236 (1996).

5. Seifert, G. Tight-binding density functional theory: An approximate Kohn-Sham DFT scheme. *J. Phys. Chem. A* **111,** 5609–5613 (2007).

6. Aradi, B., Hourahine, B. & Frauenheim, T. DFTB+, a sparse matrix-based implementation of the DFTB method. *J. Phys. Chem. A* **111,** 5678–5684 (2007).

7. Gaus, M., Goez, A. & Elstner, M. Parametrization and benchmark of DFTB3 for organic molecules. *J. Chem. Theory Comput.* **9,** 338–354 (2013).

8. Hutter, J., Iannuzzi, M., Schiffmann, F. & Van de Vondele, J. atomistic simulations of condensed matter systems. *Wiley Interdiscip. Rev. Comput. Mol. Sci.* **4,** 15–25 (2014).

9. Becke, A. Density-functional exchange-energy approximation with correct asymptotic narrier. *Phys. Rev. A* **38,** 3098–3100 (1988).

10. Lee, C., Yang, W. & Parr, R. G. Development of the Colle-Salvetti correlation-energy formula

into a functional of the electron density. *Phys. Rev. B* **37,** 785–789 (1988).

11. Vandevondele, J. *et al.* Quickstep: Fast and accurate density functional calculations using a mixed Gaussian and plane waves approach. *Comput. Phys. Commun.* **167,** 103–128 (2005).

12. VandeVondele, J. & Hutter, J. Gaussian basis sets for accurate calculations on molecular systems in gas and condensed phases. *J. Chem. Phys.* **127,** 114105 (2007).

13. Goedecker, S., Teter, M. & Hutter, J. Separable dual-space Gaussian pseudopotentials. *Phys. Rev. B* **54,** 1703–1710 (1996).

14. Hartwigsen, C., Goedecker, S. & Hutter, J. Relativistic separable dual-space Gaussian pseudopotentials from H to Rn. *Phys. Rev. B* **58,** 3641–3662 (1998).

15. Krack, M. Pseudopotentials for H to Kr optimized for gradient-corrected exchange-correlation functionals. *Theor. Chem. Acc.* **114,** 145–152 (2005).

16. Bayly, C. I., Cieplak, P., Cornell, W. D. & Kollman, P. A. A well-behaved electrostatic potential based method using charge restraints for deriving atomic charges: The RESP model. *J. Phys. Chem.* **97,** 10269–10280 (1993).

17. Martyna, G. J. & Tuckerman, M. E. A reciprocal space based method for treating long range interactions in *ab initio* and force-field-based calculations in clusters. *J. Chem. Phys.* **110,** 2810–2821 (1999).

18. Dzieciuch, M. *et al.* Pegylated liposomes as carriers of hydrophobic porphyrins. *J. Phys. Chem. B* **119,** 6646–6657 (2015).

19. Peeks, M. D., Neuhaus, P. & Anderson, H. L. Experimental and computational evaluation of the barrier to torsional rotation in a butadiyne-linked porphyrin dimer. *Phys. Chem. Chem. Phys.* **18,** 5264–5274 (2016).

20. Humphrey, W., Dalke, A. & Schulten, K. VMD: Visual molecular dynamics. *J. Mol. Graph.* **14,** 33–38 (1996).

21. A, K. Topotools: Release 1.7. (2016).

22. Jewett, A. I., Zhuang, Z. & Shea, J.-E. Moltemplate a Coarse-Grained Model Assembly Tool. *Biophys. J.* **104,** 169a (2013).

23. Lee, J. H. A Study on a Boron-Nitride Nanotube as a Gigahertz Oscillator. *J. Korean Phys. Soc.* **49,** 172–176 (2006).

24. Tuckerman, M. E., Alejandre, J., López-Rendón, R., Jochim, A. L. & Martyna, G. J. A Liouville-operator derived measure-preserving integrator for molecular dynamics simulations in the isothermal-isobaric ensemble. *J. Phys. A. Math. Gen.* **39,** 5629–5651 (2006).

25. Shinoda, W., Shiga, M. & Mikami, M. Rapid estimation of elastic constants by molecular dynamics simulation under constant stress. *Phys. Rev. B* **69,** 16–18 (2004).

26. Martyna, G. J., Tobias, D. J. & Klein, M. L. Constant pressure molecular dynamics algorithms. *J. Chem. Phys.* **101,** 4177–4189 (1994).

27. Marrink, S. J., Risselada, H. J., Yefimov, S., Tieleman, D. P. & De Vries, A. H. The MARTINI force field: Coarse grained model for biomolecular simulations. *J. Phys. Chem. B* **111,** 7812–7824 (2007).

28. De Jong, D. H. *et al.* Improved parameters for the martini coarse-grained protein force field. *J. Chem. Theory Comput.* **9,** 687–697 (2013).

29. Gobbo, C. *et al.* MARTINI model for physisorption of organic molecules on graphite. *J. Phys. Chem. C* **117,** 15623–15631 (2013).

30. Debnath, A., Wiegand, S., Paulsen, H., Kremer, K. & Peter, C. Derivation of coarse-grained

simulation models of chlorophyll molecules in lipid bilayers for applications in light harvesting systems. *Phys. Chem. Chem. Phys.* **17,** 22054–22063 (2015).

31. Miller, T. F. *et al.* Symplectic quaternion scheme for biophysical molecular dynamics. *J. Chem. Phys.* **116,** 8649–8659 (2002).

32. Morley, D. O. *et al.* A Coarse-Grained Model for Free and Template-Bound Porphyrin Nanorings. *J. Phys. Chem. A* **121,** 5907–5920 (2017).
